# Development of enterovirus transencapsidation assays as tools to understand viral entry

**DOI:** 10.1099/jgv.0.002196

**Published:** 2025-12-17

**Authors:** Philippa K. Hall, Natasha Cassani, Eero V. Hietanen, Ana Carolina Gomes Jardim, David J. Rowlands, Natalie J. Kingston, Nicola J. Stonehouse

**Affiliations:** 1 Astbury Centre for Structural Molecular Biology, School of Molecular and Cellular Biology, Faculty of Biological Sciences, University of Leeds, Leeds, UK; 2 Laboratory of Antiviral Research, Institute of Biomedical Science - ICBIM, Federal University of Uberlândia - UFU, Uberlândia, MG, Brazil

**Keywords:** enterovirus (EV), infection, replicon, transencapsidation (TE) assays, viral entry

## Abstract

Enteroviruses (EVs) are globally important human and animal pathogens that cause a diverse spectrum of diseases, ranging from febrile illness to paralysis. Despite decades of research, parts of the EV lifecycle remain poorly understood. Replicons, in which reporter genes replace the structural protein-coding region, have proved useful for the study of EV biology. However, it is not possible to study the molecular mechanism(s) of entry, capsid uncoating and genome release without the production of virus particles. To utilize the benefits provided by replicons for the study of viral cell entry, it is necessary to supply the structural proteins separately. Here, we present an EV transencapsidation (TE) system in which reporter replicons are introduced into cells that are modified to express viral structural proteins. The nascent replicons are packaged to form virus particles containing fluorescent or luminescent replicon genomes. This enables the real-time assessment of EV entry and replication through quantification of fluorescence using live-cell imaging. We demonstrate that these TE particles are biologically accurate proxies for EVA71 virions and show their utility for the study of EV entry, uncoating and replication. Additionally, we demonstrate the use of TE particles as platforms for drug discovery and immunological screening, applicable to the development of antiviral therapeutics and the assessment of immunization outcomes.

## Introduction

Enteroviruses (EVs) pose a significant threat to public health systems globally, with an estimated 10–15 million symptomatic infections occurring annually in the USA alone Centers for Disease Control and Prevention (CDC, 2024). While disease can be mild and self-limiting, life-threatening complications, including severe myocarditis, brainstem encephalitis and multiple organ failure, can occur, with neonates particularly vulnerable to these disorders [1].

The EV genus includes the archetypal poliovirus (PV), in addition to EVA71, EVD68, Coxsackieviruses, echoviruses and rhinoviruses (RVs). EVA71, a leading cause of hand, foot and mouth disease, has become endemic to the Asia-Pacific region, with major outbreaks occurring every 3–4 years [2]. A number of fatal EV outbreaks have also occurred across Europe, including echovirus 11 [3] and Coxsackievirus B in the UK [4]. Additionally, there have been biennial outbreaks of EVD68, a cause of acute flaccid myelitis [5,7], occurring globally since 2014 [8,11], and both EVD68 and EVA71 have recently been listed as high-priority pathogens by the UK Health Security Agency (UKHSA) [12]. Climate change is predicted to increase the intensity of future EV outbreaks. Indeed, one model suggests that increased global temperatures and seasonal warming could increase the size of outbreaks by up to 40%, highlighting the mounting disease burden of EVs [13]. Vaccination has been responsible for the dramatic reduction in cases of poliomyelitis worldwide. Although vaccines against EVA71 are used in the Asia-Pacific region, these are not globally available [14]. Furthermore, there are no commercially available antiviral agents against any EVs, emphasizing the critical need to develop antiviral strategies.

EVs are non-enveloped, single-stranded, positive-sense RNA viruses belonging to the *Picornaviridae* family. The genome is ~7.5 kb in length and encodes a long polyprotein, which is translated from a single ORF flanked by UTRs. Following translation initiation from an internal ribosome entry site in the 5′ UTR, the polyprotein undergoes co- and post-translational processing. This series of *cis*- and *trans*-cleavage events is mediated by two viral proteinases, 2A_pro_ and 3C_pro_, or the functional precursor 3CD_pro_, and yields a number of functional intermediates and mature products [15,16]. The precursor P1 is processed into three structural proteins, VP0, VP3 and VP1, which assemble in the presence of the genome to form a viral particle [17,21]. Following a number of conformational changes, which stabilize the particle, VP0 is cleaved into VP4 and VP2, generating an infectious virion [22,24].

Following cell attachment, EVs enter the endocytic pathway, where the genome is released into the cytosol across the endosomal membrane, most likely via a pore formed by the structural protein VP4 [25,26]. The precise molecular mechanism(s) of EV entry, uncoating and genome release are incompletely understood, highlighting the need to develop complementary strategies to better understand these processes.

Transencapsidation (TE) systems have been described for EVs, initially in 1998, where sub-genomic replicons were packaged by superinfecting virus, and expression of the reporter signal could be blocked by neutralizing antibodies [27]. This system does not improve biosafety, as infectious helper virus is required to provide structural proteins. Similar work has used several methods to generate TE particles [28], including providing structural proteins from a T7-driven DNA clone and transfecting with replicon RNA to allow the formation of infectious TE particles. These early systems showed that homologous or heterologous viral structural proteins (or ‘helper viruses’) can package reporter genomes *in trans* to produce functional pseudovirions. As the structural proteins are absent from the encapsidated replicon genome, this approach provides significantly improved levels of biosafety.

More recent studies have generated TE particles from cells that have been transduced to stably express viral P1. This has included adapting the TE system to monitor EV replication in real-time at single-molecule resolution [29]. Other TE systems expressed N-terminal EGFP linked by a 2A site to the P1 sequence. Although the presence of this 2A sequence may interfere with the natural myristoylation of the N-terminus of VP4, TE particles were produced following RNA transfection [30].

Advances in fluorescent reporter technologies and real-time readout systems have enabled us to adapt and advance TE systems to facilitate easy monitoring of the dynamics of viral entry and replication. We electroporated viral replicon RNA into cells expressing structural proteins derived from a DNA plasmid, to generate functional TE particles from the cognate replicon/capsid protein pair.

Using these tools, we established that TE particles are a suitable and safe proxy for EVA71 studies, demonstrating biological properties akin to WT virus. Moreover, we show that the TE system can be utilized as a multifunctional platform for accurate and rapid compound and immunological screening and as a tool to better understand EV entry, enabling a sensitive quantification of entry and uncoating kinetics in real-time.

## Methods

### Virus recovery

EVA71 (genogroup B2) virus was recovered from *in vitro*-transcribed RNA as previously described [31]. Briefly, a WT EVA71-encoding plasmid was linearized and purified with the Monarch PCR and DNA Cleanup Kit (NEB). Full-length genomic RNA was synthesized using the T7 RiboMax Express Large-Scale RNA Production System (Promega) and purified using the RNA Clean and Concentrator Kit (Zymo Research, USA). The purified RNA was electroporated into HeLa cells within 4 mm cuvettes at 260 V for 25 ms (square wave). Cells were incubated at 37 ˚C in 5% CO_2_, and samples were harvested 18 h post-electroporation.

### Replicon assays

HeLa cells were seeded into 96-well plates (2.5×10^4^ cells per well) and incubated at 37 ˚C overnight. The media was replaced with phenol red-free Dulbecco's Modified Eagle Medium (DMEM), and the cells were transfected with replicon RNA (167 ng per well) using Lipofectamine 2000 (Thermo Fisher). The fluorescence produced by the cells was monitored hourly for 24 h using an Incucyte S3 system (Sartorius) through quantification of both total green and red object-integrated intensity and fluorescent count per image. For analysis, fluorescence was defined using the GNN control, as a surface fit with a threshold of 2.00 Green Corrected Units (GCU) and −40 edge sensitivity (edge split) using an Incucyte S3 system. Luciferase assays were performed using the Bright-Glo Luciferase Assay System (Promega) following the manufacturer’s protocol and quantified using the GloMax Discover (Promega). Intracellular genome quantification from replicon transfection was not possible due to large amounts of input RNA (~10^14^ copies), far exceeding that which is generated during replication.

### Production of TE particles

HeLa cells were transfected in suspension (1×10^7^ cells and 25 µg plasmid DNA per T175 flask). DNA plasmids were generated using the pCAGGS vector, expressing the full-length P1 region or an empty-vector control. Transfections were performed at a 1 : 3 (w/w) ratio with polyethylenimine before being incubated at 37 ˚C in 5% CO_2_ for 48 h. P1 expression was validated by western blot (Fig. S1, available in the online Supplementary Material). Cells were subsequently electroporated with *in vitro-*transcribed replicon RNA as described above. Supernatant samples were collected after 18 h and clarified by centrifugation at 4,000 relative centrifugal force (rcf).

### TE assay and EC_50_ screens

HeLa cells were seeded into 96-well plates (2.5×10^5^ cells per millilitre) and incubated at 37 ˚C overnight in 5% CO_2_. The media was replaced with phenol red-free DMEM, and cells were infected with 10 µl of TE inoculum. The fluorescence produced from the transencapsidated replicons was monitored (as above) using an Incucyte S3 system (Sartorius). EC_50_ screens were performed with the seeded cells pre-treated for 30 min with the compounds enviroxime or rupintrivir at concentrations ranging from 100 to 0.01 µM, or bafilomycin A1 at concentrations ranging from 2 µM to 2 nM. The compounds were maintained in the media following the addition of the TE inoculum. All EC_50_ values were determined at 24 h, with total green object-integrated intensity normalized to the DMSO control and presented as a percentage. TE particles bearing the luciferase reporter gene were treated in the same way and assessed after 24 h.

### Cell viability assay

HeLa cells were seeded into 96-well plates (2.5×10^5^ cells per millilitre) and incubated at 37 ˚C overnight in 5% CO_2._ The media was replaced with concentrations of enviroxime and rupintrivir ranging from 100 to 0.01 µM, and bafilomycin A1 (2 µM to 2 nM), and cells were incubated for 24 h. CellTiter 96 AQueous One Solution Cell Proliferation Assay (Promega) was used following the manufacturer’s instructions, and the absorbance was quantified using the GloMax Discover (Promega).

### Sucrose gradients

Clarified supernatants from virus and TE particle recoveries were loaded onto discontinuous 15–45% sucrose gradients and centrifuged at 50,000 rcf for 12 h. Following centrifugation, seventeen 1 ml fractions were collected (top-down) (as described in Kingston *et al.* [32]).

### RT-qPCR

Reverse transcription quantitative polymerase chain reaction (RT-qPCR) was performed as previously described [22]. Briefly, samples collected from the sucrose gradient fractions were diluted ten-fold in nuclease-free water with RNAsecure (Thermo Fisher) added. Samples were heated for 10 min at 60 ˚C to facilitate RNA release and processed in a one-step RT-qPCR (Promega) following the manufacturer’s instructions.

### TCID_50_ assay

Ten-fold serial dilutions of virus were made in DMEM supplemented with 2% FBS, and 100 µl per well of each dilution was added to Vero cells seeded in 96-well plates (1×10^4^ cells per well). Following a 5-day incubation at 37 ˚C in 5% CO_2_, cells were fixed with 4% formaldehyde and incubated at room temperature for a minimum of 1 h before staining with crystal violet solution. The calculation was determined by the Reed–Muench method.

### Blind passage of TE particles

HeLa cells were seeded into six-well plates (1×10^5^ cells per millilitre) and incubated at 37 ˚C in 5% CO_2_ overnight. The media was replaced, and cells were infected with 10 µl of TE inoculum. Following a 3-day incubation at 37 ˚C in 5% CO_2_, cells were harvested and clarified. Clarified supernatant was used for subsequent cycles of passage. A total of three passages were completed. TE assays were performed (as above) with the passaged supernatants.

### Validation of packaged reporter RNA

Integrity of the RNA packaged in the TE particles was validated by Nanopore sequencing. RNA extractions were carried out following standard protocols [20]. The extracted RNA was reverse-transcribed using the Transcriptor First Strand cDNA Synthesis Kit (Roche), and subsequently PCR amplified with primers spanning a 951-nt section covering the reporter gene. A sequencing library was prepared using the Native Barcoding 24 Kit (SQK-NBD114-24; Oxford Nanopore Technologies) following the manufacturer’s instructions. The prepared library was loaded onto an R10.4.1 flow cell (FLO-MIN114; Oxford Nanopore Technologies) and sequenced on a MinION Mk1C platform with MinKNOW v25.05.14 (Oxford Nanopore Technologies).

Basecalling was performed with Dorado v0.8.2 (Oxford Nanopore Technologies) using the super-high-accuracy model (dna_r10.4.1_e8.2_400bps_sup@v5.0.0). The quality-controlled and basecalled reads were subsequently aligned to the reference sequence of the amplified target region using minimap2 v2.3, using the preset for Nanopore data [33]. Additional required file manipulation was undertaken using samtools v1.21 [34]. Variant calling [checking for insertions, deletions and single nucleotide polymorphisms (SNPs)] was carried out in Geneious Prime 2025.2.2 (http://www.geneious.com) with the default settings, as well as by adjusting the variant-frequency threshold lower to 5% to check for possible low-frequency variants.

## Results

### Generation of fluorescent reporter TE particles

**Fig. 1.**
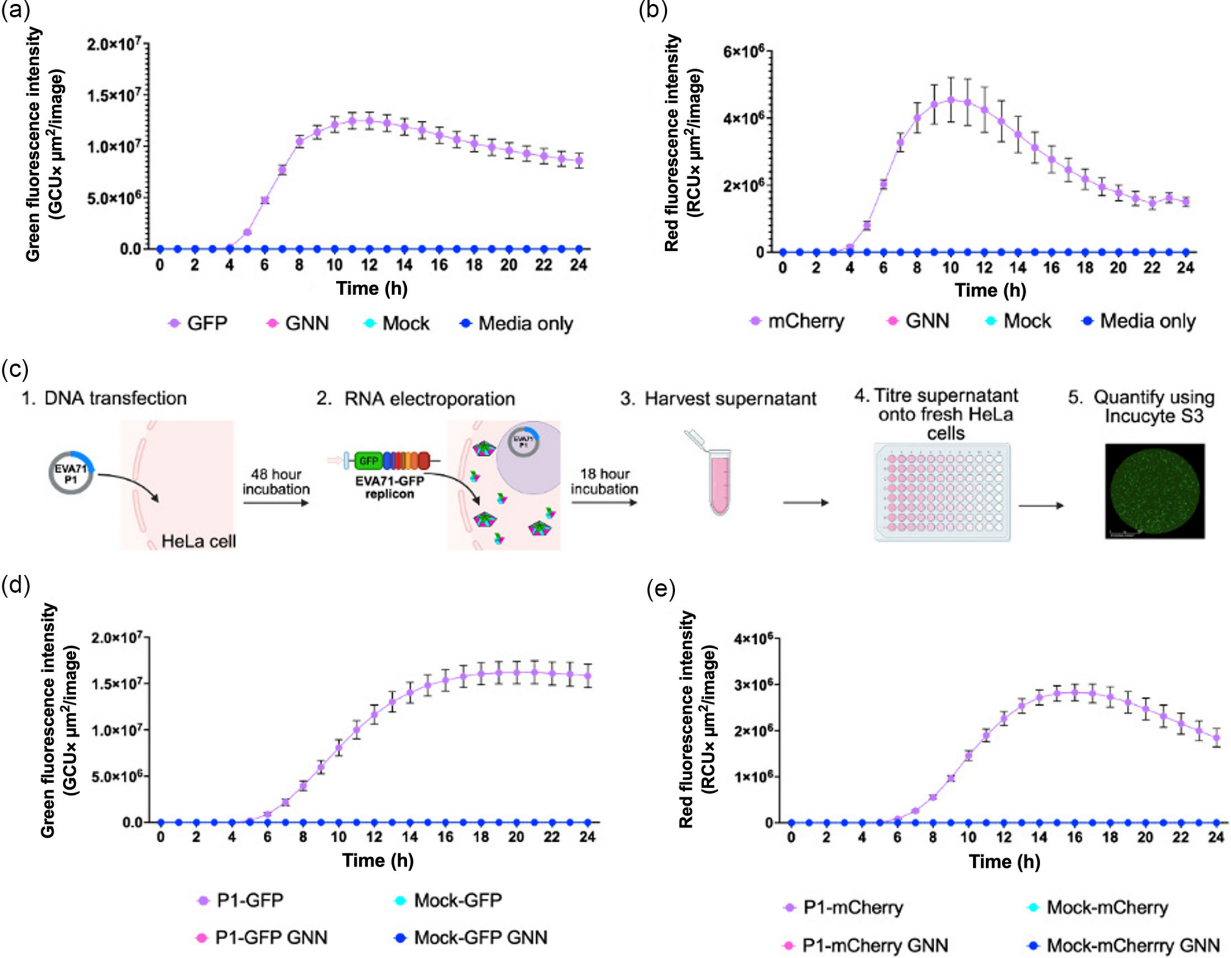
Generation of fluorescent reporter-containing TE particles. Replication kinetics of (a) EVA71-GFP and (b) EVA71-mCherry replicons over 24 h after transfection into HeLa cells, alongside GNN controls for input translation. (**c**) Diagrammatic representation of the protocol used to generate and assess TE particles (BioRender). (**d**) Kinetics of P1-GFP and (e) P1-mCherry TE infection assays. HeLa cells were infected with TE particles alongside mock controls. The production of fluorescence was monitored across 24 h using an Incucyte S3. Total green or red object integrated intensity is presented as green or red fluorescence. Assays were performed in triplicate, graphed as mean ± sem (*n*=3 in triplicate).

To monitor the replication of EVA71 fluorescent replicons, cells were transfected with *in vitro*-transcribed replicon RNA, and the production of fluorescence was measured over time. Replicons in which the RNA-dependent RNA polymerase incorporated an active-site mutation (GNN) were included to assess the levels of input RNA translation (Fig. 1a, b). As expected, WT replicons were replication-competent, with peak fluorescence detected ~10 h post-transfection. Fluorescence intensities were slightly different, with ~1.2×10^7^ GCU× µm^2^ per image for the GFP replicon and ~4×10^6^ red corrected units (RCU) × µm^2^ per image for the mCherry replicon. Minimal fluorescence produced by input translation in the replication-deficient GNN control was used to define background fluorescence.

To determine the potential for the (separately produced) viral structural proteins to package these replicons, we expressed the EVA71 P1 structural protein precursor from an independently introduced DNA construct. Cells expressing the P1 protein were then electroporated with *in vitro*-transcribed RNA of WT or GNN replicons, alongside mock-transfected cells (empty vector) as controls (Fig. 1c). Culture supernatants were clarified and used to infect naïve HeLa cells, and fluorescence was monitored via an Incucyte S3 live-cell imaging system (Figs 1d, e and S2).

Cells infected with TE particles (P1-GFP and P1-mCherry) produced maximal fluorescence around 16 h post-infection (Fig. 1d, e), with maximum fluorescence intensities comparable to the replicon assays (Fig. 1a, b). The EVA71-GFP reporter system showed higher average fluorescence intensity than the mCherry equivalent in both replicon and TE systems, with recovered P1-GFP TE particles typically yielding ~7.2×10^6^ infectious units per millilitre (Fig. S3). An infection curve with varying amounts of TE particle inoculum was carried out, and a 10 µl inoculum per well of P1-GFP TE-recovered supernatant was selected as the appropriate dose for future analyses (Fig. S4). No fluorescence was detected above background levels in any of the control wells (Mock or GNN), consistent with the requirement for functional polymerase and nascent genome synthesis for viral assembly. The integrity of the replicon RNAs within the TE particles was assessed for the presence of deletions, insertions or SNPs in the reporter gene sequence by nanopore sequencing. No such mutations were detected within the encapsidated reporter RNA. Serial passage was performed to assess the biosafety of the TE particles, and no recovery of infectious virus was detected over three passages (Fig. S5). Together, these results indicate that cognate P1 supplied in trans can package fluorescent replicons, making this a suitable method to generate particles capable of infecting susceptible cells.

### Generation of luciferase reporter TE particles

**Fig. 2.**
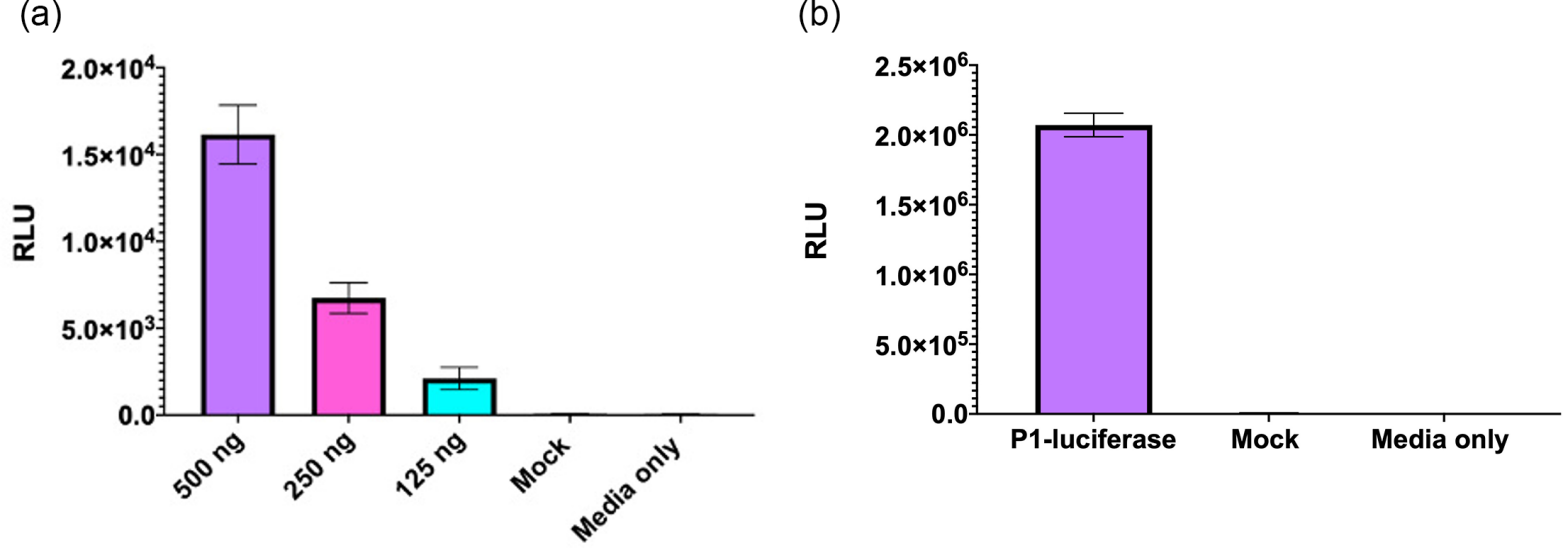
Generation of luciferase-containing TE particles. (**a**) Luciferase signal generated by the EVA71 fLuc replicon 24 h post-transfection. Luciferase activity was measured and graphed as mean ± sem (*n*=3 in triplicate). (**b**) Cells infected with fLuc-TE particles were assessed for the presence of fLuc after 24 h. Graphed as mean ± sem (*n*=3 in triplicate).

Replicons in which the fluorescent reporter gene was replaced by a firefly luciferase (fLuc) gene were produced to understand the general utility of the system. This modification allows the TE platform to be used without the requirement for a real-time fluorescent read-out system. To assess the function of these replicons, cells were transfected with the replicon RNA, and the presence of fLuc was measured 24 h post-transfection (Fig. 2a). A dose-dependent relationship was observed, with cells transfected with 500 ng of RNA demonstrating significantly higher signals than the cells transfected with 250 or 125 ng of RNA. We then explored whether the fLuc replicon was also compatible with our TE platform (Fig. 2b).

Cells infected with the luciferase TE particles generated ~2.0×10^6^ relative light units (RLU) after 24 h, indicating that the TE platform can be utilized to package an fLuc replicon. Interestingly, the cells infected with the luciferase TE particles consistently generated higher (~2 log) signals than replicon-transfected cells. To determine if this difference was due to luciferase degradation, samples were assessed for full-length luciferase by western blot at 6 and 24 h post-replicon transfection and TE infection. While the infected sample produced luciferase detectable at 6 h post-transfection, both transfected and infected samples showed the presence of fLuc at 24 h. In both cases, minimal degradation was noted for the fLuc reporter, and there was no significant difference in band intensity (Fig. S6).

### Characterization of TE particles and comparison with EVA71 virus particles

**Fig. 3.**
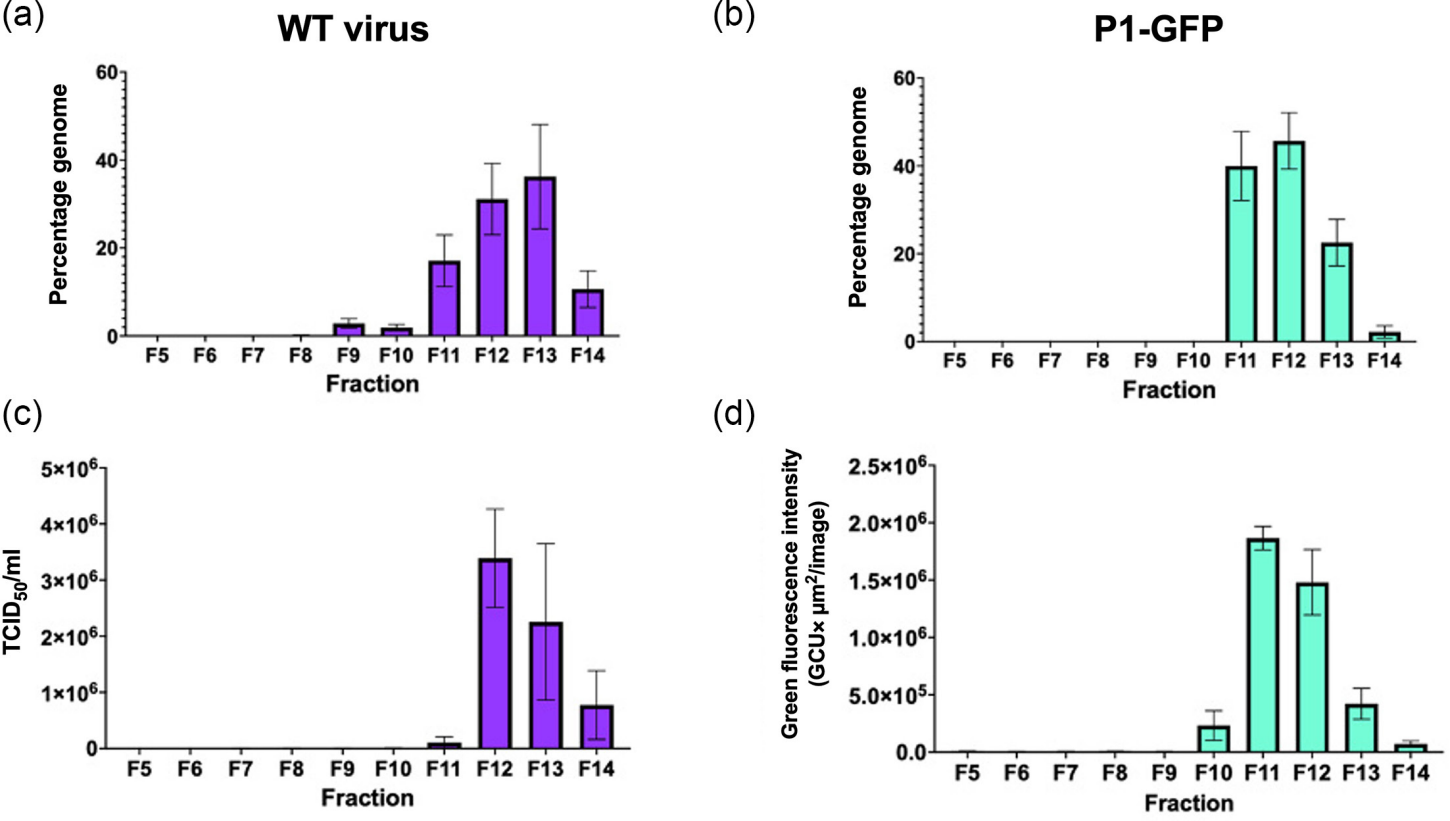
Characterization of TE particles and virus. Samples of WT EVA71 and P1-GFP TE particles were recovered directly from *in vitro*-transcribed RNA electroporated into HeLa cells and separated on 15–45% sucrose gradients. RT-qPCR was used to determine the presence of viral RNA in (a) WT EVA71 virus and (b) P1-GFP, with total genome content presented as the percentage of genome detected within the gradient, graphed as mean ± sem (*n*=3 in duplicate). (**c**) Infectious titre of WT virus and (d) P1-GFP particles (24 h timepoint), with total green object integrated intensity presented as green fluorescence, graphed as mean ± sem (*n*=3).

To compare the molecular properties of TE particles and virus, clarified supernatant samples were recovered in parallel and separated through sucrose gradients, which were then fractionated from the top of the gradient. Gradient fractions were assessed for infectious titre by TCID_50_ assay or fluorescence reporter assay, and for the presence of genome by RT-qPCR. Consistent with previous observations [22], the majority of genome was detected in fractions 12 and 13 in the WT virus sample (Fig. 3a). However, the TE particles showed peak genome in fractions 11 and 12 (Fig. 3b). Consistently, peak infectious titres for WT virus were in fraction 12 (Fig. 3c), and TE particles showed peak infectivity in fraction 11 (Fig. 3d). No genome was detected in the GNN control (Fig. S7). This sedimentation difference may be attributable to the smaller mass of the GFP replicon in comparison with the EVA71 genome. Collectively, this indicates that TE particles are formed and are able to package genomes in a manner similar to WT virus.

### TE system to study entry

**Fig. 4.**
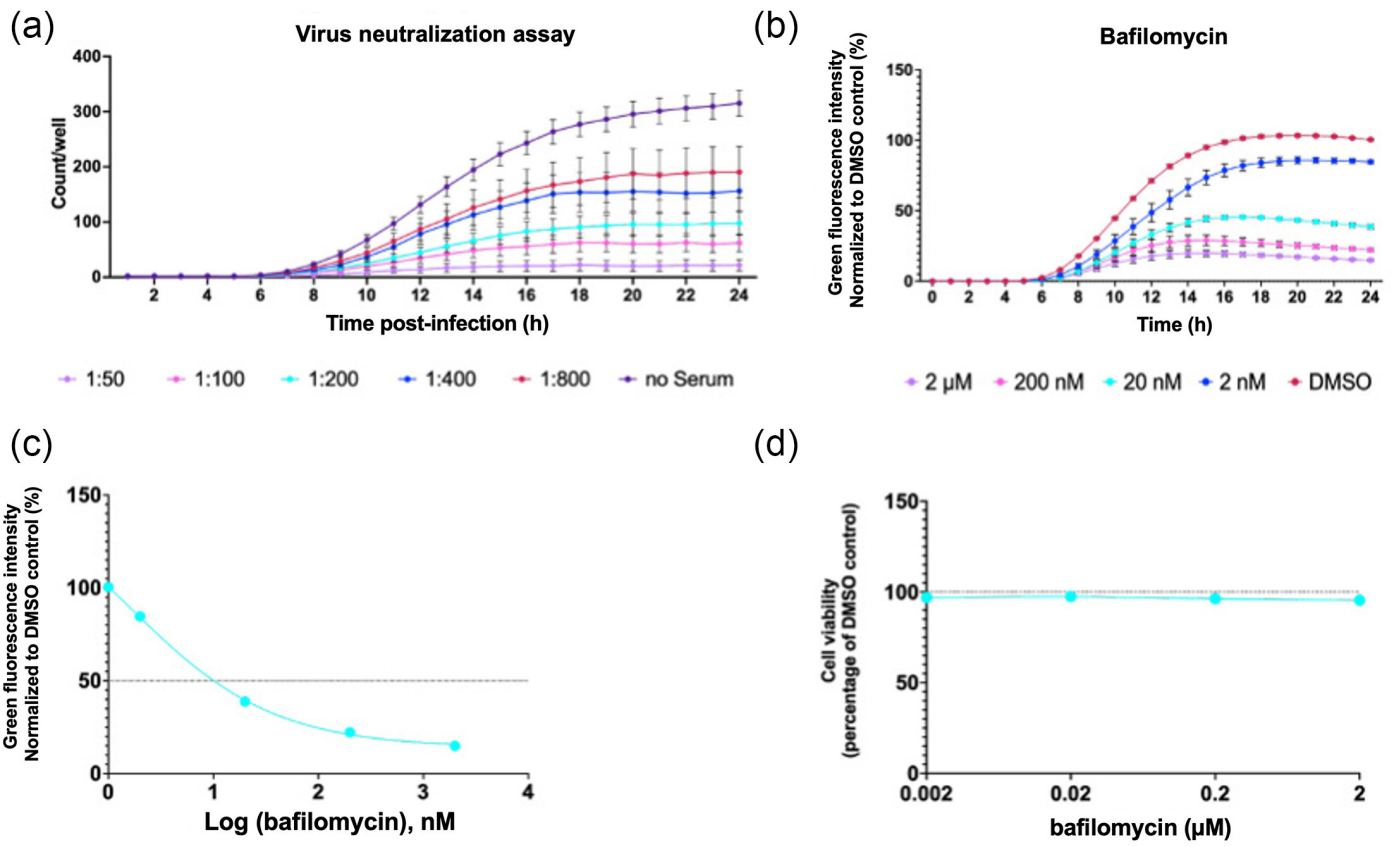
Entry inhibition. (**a**) Virus neutralization assay. GFP TE particles were incubated with rat anti-EVA71 immune serum for 1 h at the indicated dilutions. The mixtures were added to pre-seeded HeLa cells, and the fluorescent counts quantified 24 h post-inoculation using an Incucyte S3. (**b**) HeLa cells were treated with bafilomycin A1 or DMSO and infected with P1-GFP TE particles. (**c**) EC_50_ curve of bafilomycin A1. EC_50_ values were calculated in GraphPad. Total green object integrated intensity is presented as green fluorescence, normalized to the DMSO control at 24 h. (**d**) Cell viability with an MTS assay after incubation with a concentration series of bafilomycin A1, normalized to the DMSO control at 24 h. Assays were performed in triplicate, graphed as mean ± sem (*n*=3 in triplicate). MTS is the standard nomenclature for this assay, the chemical used is: (3-(4,5-dimethylthiazol-2-yl)-5-(3-carboxymethoxyphenyl)-2-(4-sulfophenyl)-2H-tetrazolium).

The requirement for the viral structural proteins in this platform enables investigation of their roles during viral entry and capsid uncoating. To further validate the TE system as a tool to study EV entry, we carried out antibody-mediated virus neutralization assays and bafilomycin-based entry inhibition assays. P1-GFP TE particles were incubated with dilutions of immune sera at 1:50–1:800, and following 1 h incubation at 37 ˚C, TE particles were added to HeLa cells. Fluorescence was assessed hourly over 24 h post-infection. A dose-dependent relationship was observed, with the 1:50 dilution of immune sera producing the lowest fluorescent counts per well (Fig. 4a). As a proof of concept, this indicates that the P1-GFP TE particles are susceptible to neutralizing antibodies, as expected, and the system can be utilized to assess antibody-mediated virus neutralization within a 24 h timeframe.

Bafilomycin (BafA1), a specific inhibitor of vacuolar proton ATPases [35], is commonly employed as a tool to understand the entry pathways of a number of viruses [36,39]. To determine if the TE system could be utilized to understand EV entry kinetics, HeLa cells were pretreated with BafA1 at concentrations of up to 2 µM and infected with GFP TE particles, with BafA1 remaining present in the media. The fluorescence was assessed across 24 h post-infection and normalized to the DMSO solvent control to determine the production of fluorescence.

BafA1 treatment reduced the fluorescence generated in a concentration-dependent manner, with 2 µM BafA1 decreasing fluorescence intensity to ~15% of the DMSO control (Fig. 4b). The EC_50_ was determined to be 0.001 µM at 24 h (Fig. 4c), suggesting that BafA1 treatment is able to inhibit the entry of the TE particles. Critically, no cytotoxic effect was observed at any of the concentrations tested (Fig. 4d).

### TE system to study replication

To establish if the TE system could be utilized as a tool to study EV replication and function as a platform for antiviral compound screening, we performed inhibition assays with previously validated EV antiviral compounds. Enviroxime, a compound that has been shown to target the 3A protein of PV and RV [40], and rupintrivir, an inhibitor of RV 3C protease [41], were selected as examples in this study. To determine the effects of enviroxime and rupintrivir in TE particle infection studies, HeLa cells were treated with each compound at 0.01–100 µM. After 30 min of pre-treatment, cells were infected with P1-GFP, and the compounds remained present in the media. The fluorescence generated was quantified across 24 h post-infection and normalized to the DMSO control at 24 h.

**Fig. 5.**
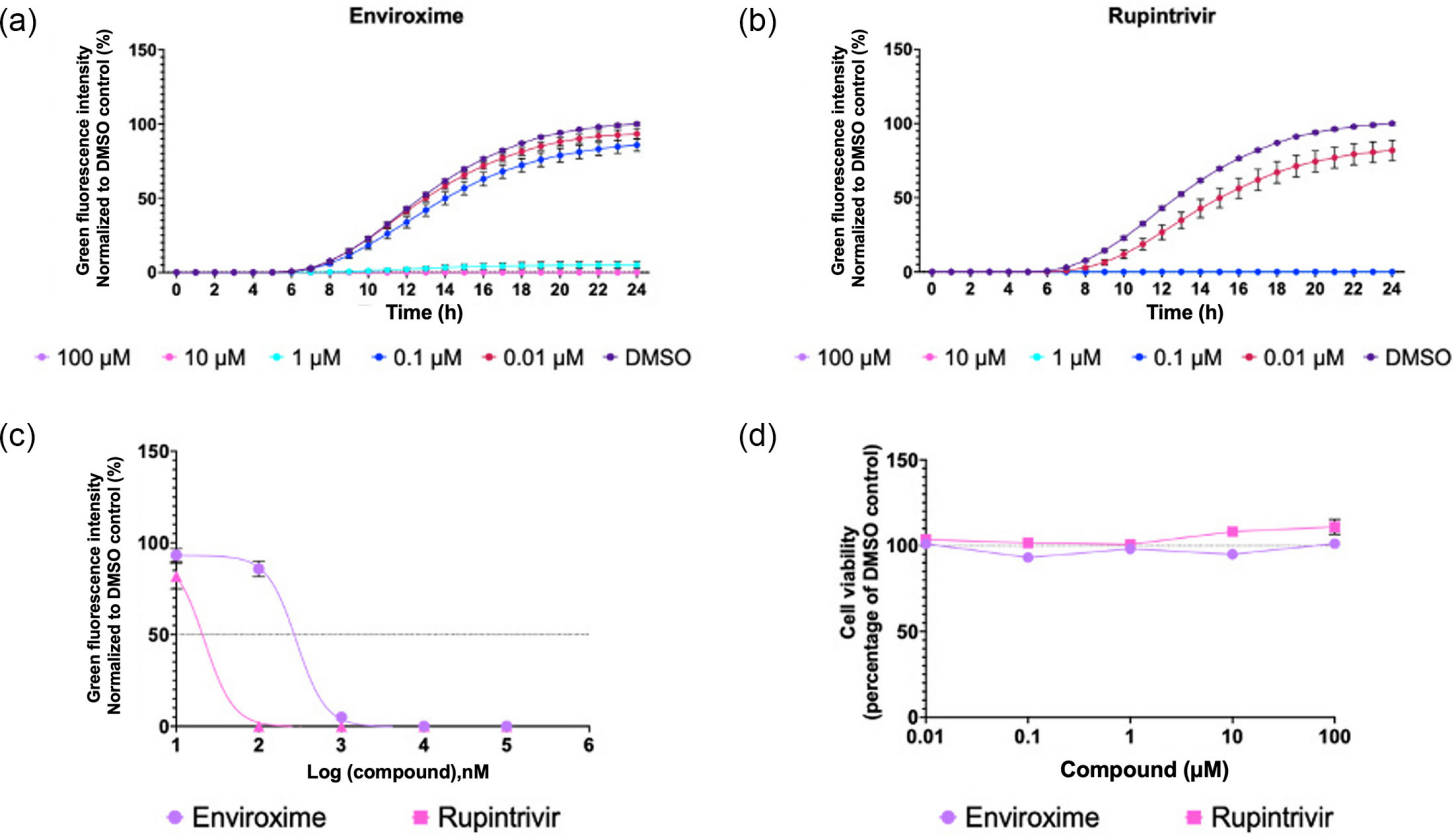
Replication inhibition. HeLa cells were treated with (a) enviroxime, (b) rupintrivir or DMSO and infected with P1-GFP TE particles. Fluorescence measurements were normalized to a DMSO control at 24 h. (**c**) EC_50_ curves of enviroxime and rupintrivir. EC_50_ values were calculated in GraphPad. Total green object integrated intensity is presented as green fluorescence, normalized to the DMSO control at 24 h. (**d**) MTS assay measuring cell viability at 24 h in the presence of compound, normalized to DMSO. Assays were performed in triplicate, graphed as mean ± sem (*n*=3 in triplicate). MTS is the standard nomenclature for this assay, the chemical used is: (3-(4,5-dimethylthiazol-2-yl)-5-(3-carboxymethoxyphenyl)-2-(4-sulfophenyl)-2H-tetrazolium).

Higher concentrations of both enviroxime (Fig. 5a) and rupintrivir (Fig. 5b) resulted in a reduced level of fluorescence, with EC_50_ values of 0.228 and 0.019 µM, respectively (Fig. 5c). Rupintrivir therefore appears more effective than enviroxime at inhibiting EVA71 replication. Importantly, neither compound showed cytotoxic effects at any concentration tested (Fig. 5d). Together, this panel of assays validates the potential for TE particles to function as reliable reporter systems for the assessment of compounds against EVs.

## Discussion

The development of tools with improved biosafety is beneficial for the study of viral functions and the development of new antiviral therapeutics to combat the global public health threat EVs pose is urgently required. Here, we present a sensitive TE system whereby the P1 structural proteins are provided in trans to package cognate replicons within capsid proteins to produce fluorescent or luminescent TE reporter particles, which are biologically accurate proxies for WT virions.

The fluorescent signal generated facilitates live-cell imaging, allowing entry and replication to be visualized and quantified easily in real time using an Incucyte system (Fig. 1). Alternatively, the luminescent reporter enables a highly sensitive measurement system using widely available read-out technology (Fig. 2). We have also demonstrated the general applicability of this system for the study of EV entry kinetics, assessment of inhibitory compounds and immunological responses.

The use of fluorescent and luminescent replicon systems across the *Picornaviridae* family has aided the study of viral replication, representing viable alternatives to live-virus studies with improved health and safety profiles [42,43]. However, standard replicons lack the viral structural proteins and rely on transfection protocols, which makes them unsuitable for the study of early events in the viral life cycle, such as cell entry and uncoating. The fluorescent TE assays overcome this restriction, enabling the measurement of entry kinetics through the quantification of fluorescence as a proxy. The time taken for viral uncoating to occur is reflected in the delay in maximal fluorescence of the TE infection system compared with the replicon transfection assay (Fig. 1). The cells infected with TE particles generated peak fluorescence ~16 h, whereas the cells transfected with the replicon produced maximum fluorescence ~10 h, although the maximum fluorescent intensities (Fig. 1) and counts (Fig. S2) were similar. The 6 h difference following TE infection in comparison to transfection was unexpected. Earlier reports suggest that the entry process occurs within minutes or hours of an EV infection [44,45], with one report suggesting that WT EVA71 is fully uncoated at 2 h post-infection [39]. Input translation from TE particles can be detected as early as ~10 min post-infection [29]. The time difference between TE particle infection and replicon transfection shown here is most likely due to differences in the method of intracellular genome delivery, resulting in synchronous versus asynchronous replication. While both systems target a similar number of cells (Fig. S2), replicon transfection is likely to introduce many more genome copies per cell compared with TE infection. Consequently, the initial stages of replication complex formation, host cell shut-off and subsequent translation-to-replication switches would likely occur faster, possibly contributing to the time difference to maximum fluorescence. Unlike the fluorescent constructs, which showed similar intensities of reporter gene expression whether wells were transfected or infected, a ~2 log difference in measured luciferase activity was observed between the infected and transfected wells (Fig. 2). Previous reports of luciferase reporter TE particles were not directly compared with luciferase replicon assays, thus the differences observed here may have been missed in earlier studies [28,30]. Using western blot to directly measure luciferase protein, we did not detect meaningful differences in the accumulation of full-length or cleavage products for fLuc, which may have contributed to differences in the luminescence detected (Fig. S6). What was apparent was a more rapid accumulation of luciferase signal in transfected samples, together with a signal of similar intensity at 24 h post-infection in TE-infected samples. This suggests that, despite samples transfected with replicon producing proteins more rapidly, infected cells generate higher luciferase signal at 24 h without a noticeable difference in yields of reporter protein. Although we cannot discount kinetic differences in polyprotein processing, formation of replication complexes and/or accumulation of proteases, the observed kinetic differences may reflect the nature of an optimized viral replication strategy, with the initiation of infection often derived from a single genome per cell [29]. This observation ultimately suggests that, while a useful system for studying viral replication, the conclusions drawn from replicon assays should be treated with caution unless supported by additional studies.

Comparison of the TE particles with EVA71 virions showed a minor difference in the sedimentation profiles of the particles. Peak genome levels and infectious particle titres were observed in fractions 11 and 12 for TE particles, and fractions 12 and 13 for virions (Fig. 3). As the EVA71 replicon is ~1,700 nt shorter (~550 kDa; ~10 S) than the genome of EVA71, this mass difference likely accounts for the difference in sedimentation characteristics. Additionally, the infectious nature of these particles suggests that the sedimentation differences are not due to conformational differences, as alternative particle forms, for example, uncoating intermediate states, are associated with loss of infectivity [46].

An advantage of the TE system described here is that it allows the study of virus-entry events, combined with the benefits of a sensitive reporter system and increased biosafety. Consequently, in contrast with replicon transfection assays, compounds that may target capsid protein-related functions can be screened using the TE system. Our TE assay enables the measurement of entry kinetics in concert with replication, allowing key insights into the roles of the structural proteins during EV infection. This is illustrated by the sensitivity of TE particles to BafA1 treatment and their utility in serum neutralization assays (Fig. 4). Importantly, this TE system provides a significantly reduced turnaround time compared with live-virus approaches. For example, EVA71 antibody-based virus neutralization assays require a ~5-day incubation, whereas these results were obtained within 24 h [47]. This may be of value for the development of vaccines and antiviral therapeutics against picornaviruses, and future studies aim to optimize this platform for EVD68, PV and alternative EVs.

The separation of viral capsid components from replicon reporter genomes makes the TE system useful for detailed study of the molecular events underlying uncoating and genome delivery into the host cell. Specific mutations can be readily introduced into the structural genes, and their effects on the infection process monitored using an invariable reporter genome. Future work will, therefore, include EV mutants with altered entry or uncoating phenotypes, using real-time monitoring of reporter expression as a proxy for infection rate.

Genome delivery via TE particles can be a useful and rapid route for antiviral discovery, illustrated by testing the effects of two known anti-EVA71 inhibitory compounds, enviroxime and rupintrivir, on GFP reporter replicons delivered as TE particles (Fig. 5). The inhibitory concentrations determined by this method were consistent with those determined for WT EVA71 infection. The EC_50_ of enviroxime (0.228 µM) matched published values for enviroxime against EVA71 (0.06 and 1 µM) [48,50]. Similarly, the EC_50_ of rupintrivir in our TE system (0.019 µM) is consistent with the published EC_50_s ranging from 0.009 to 0.18 µM [49,51, 52]. Together, the data suggest that the TE system is an accurate and reliable measure of EVA71 inhibition, akin to live-virus studies, validating it as a compound screening platform.

Recent developments in the design of EV reverse-genetic systems have facilitated the production of a suite of infectious clones of EVA71 incorporating novel reporter genes [53]. Despite the many advantages of these approaches, the infectious nature means that biosafety remains a serious concern, and the frequency of reporter genes incorporating mutations induced in error-prone replication limits the functionality and reliability of these systems. TE systems, however, eliminate biosafety concerns, and the input material is derived from cloned RNA and involves a single cycle of infection, thus reducing/eliminating the probability of introducing mutations. This was confirmed through the serial passage of the TE particles, demonstrating no recovery of infectious material over three passages (Fig. S8). Importantly, our TE assays did not show any reporter failure through sequence modification, demonstrating enhanced stability and consistency.

The versatility of the TE system means that many reporter genes could be utilized in this platform. Pt-GFP was chosen here as this reporter is less susceptible to processing by either of the viral proteases 2A_pro_ and 3C_pro_ [54]. We also use the mCherry reporter, although this appears more susceptible to cleavage, indicated by a reduction in fluorescence after 11 h (Fig. 1b). This is evident in both the replicon and TE assays, where the fluorescent signal generated by the cells transfected or infected with either mCherry replicon or mCherry TE particles rapidly decreases once maximal fluorescence intensity has been achieved. This contrasts with the GFP signal from replicons and GFP TE particles, which is considerably more stable. Reporter cleavage is not unexpected, with multiple viral proteases and multiple recognition sequences for proteolysis present in picornavirus genomes [54,57]. Future studies could improve the protease resistance of reporter constructs.

Together, these results validate a method to generate TE EVA71 particles incorporating fluorescent and luminescent reporter replicons and validate the system as a suitable proxy for WT EVA71 virions. We have also demonstrated the applicability of the system for the study of EV entry and as a drug and immunology screening approach, thus providing a beneficial tool and safer alternative to advance EV biology.

## Supplementary material

10.1099/jgv.0.002196 Uncited Fig. S1.

## Abbreviations

DMEM: Dulbecco's modified eagle medium
EV: enterovirus
fLuc: firefly luciferase
GCU: Green corrected units
PV: poliovirus
rcf: relative centrifugal force
RCU: red corrected units
RT-qPCR: reverse transcription quantitative Ppolymerase chain reaction
RV: rhinovirus
SNP: single nucleotide polymorphism
TE: transencapsidation
