# Supplementary material for: Development of enterovirus transencapsidation assays as tools to understand viral entry
Source: J Gen Virol. 2025 Dec 17;106(12):002196. doi: 10.1099/jgv.0.002196 (PMC12710768; doi:10.1099/jgv.0.002196)
Supplement: Uncited Fig. S1. [file jgv-106-02196-s001.pdf]

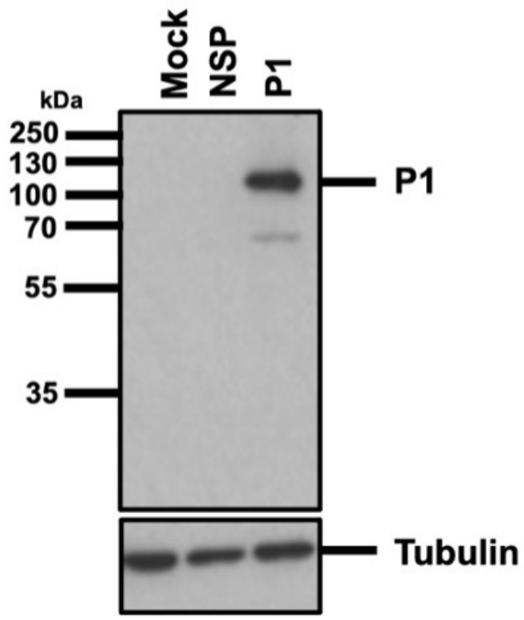


**Figure S1. Validation of P1 expression**

Validation of expression of EVA71 P1 polyprotein in HeLa cells. Anti-VP2 (mAb979) western blot of cell HeLa cell lysates after transfection with empty vector (Mock), a vector encoding an EVA71 non-structural protein (NSP), or EVA71 P1 (P1). Image edited for clarity, approximate molecular weights indicated in kDa.

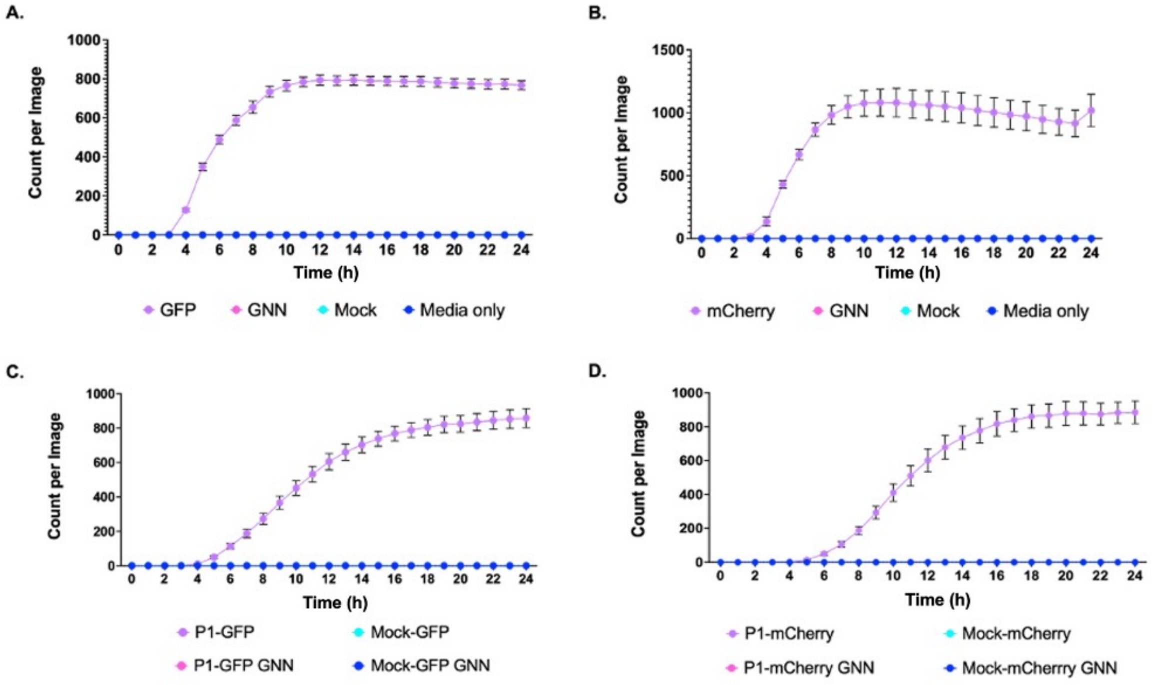


**Figure S2. Comparison of replicon and TE kinetics.**

Replication kinetics of **A)** EVA71-GFP and **B)** EVA71-mCherry over 24 hours after transfection of HeLa cells with replicon RNA, alongside replication-deficient replicon (GNN) controls. **C)** Kinetics of P1-GFP and **D)** P1-mCherry TE infection assay. HeLa cells were infected with P1-GFP TE particles alongside mock controls. The production of fluorescence was measured across 24-hours using an Incucyte S3. Assays performed in triplicate, graphed mean and ± SEM n = 3 in triplicate.

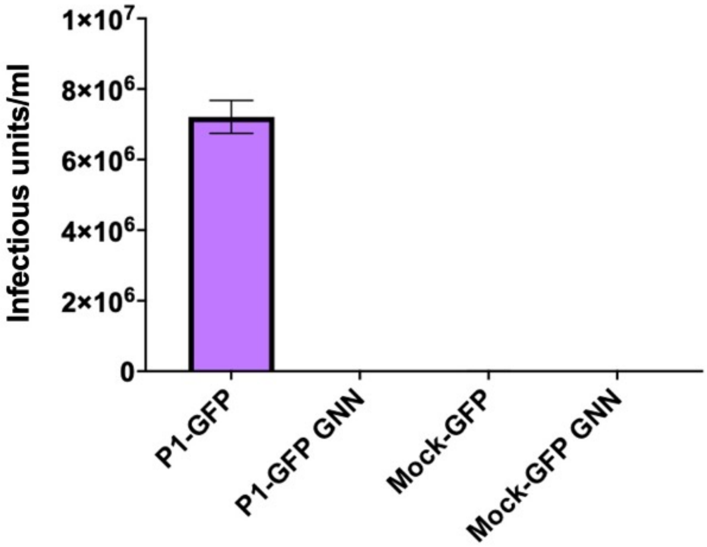


**Figure S3. Yield of TE particles**

Yield of P1-GFP TE particles from a TE infection assay at 24-hours post infection. HeLa cells were infected with TE particles alongside GNN and mock controls. The production of fluorescence was measured using an Incucyte S3. Assay performed in triplicate, graphed mean and ± SEM n = 3 in triplicate.

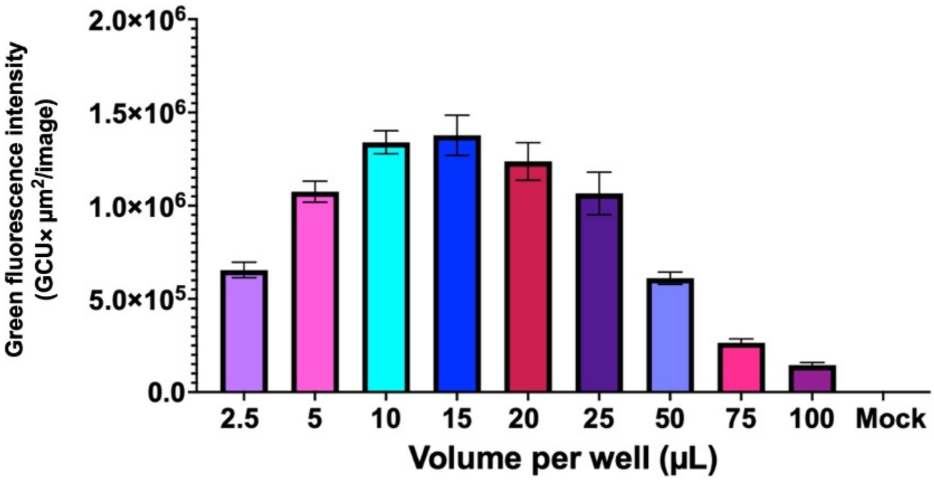


**Figure S4. TE infection curve**

HeLa cells were infected with varying amounts of P1-GFP TE particles alongside mock control. The production of fluorescence was assessed at 22-hours post infection using an Incucyte S3. Assays performed in triplicate, graphed mean and ± SEM n = 3 in triplicate.

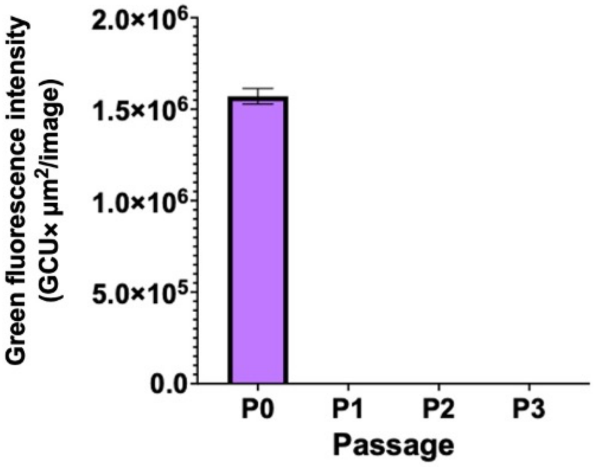


**Figure S5. Serial passage of TE particles**

Samples of P1 GFP TE particles were recovered directly from in vitro transcribed RNA electroporated into HeLa cells and used to infect pre-seeded HeLa cells for serial passage. Following three successive rounds of passage, the harvested supernatants were used for a TE infection assay. The production of fluorescence was measured at 24-hours post infection utilising an Incucyte S3 system. Assays performed in triplicate, graphed mean and ± SEM n = 3 in triplicate.

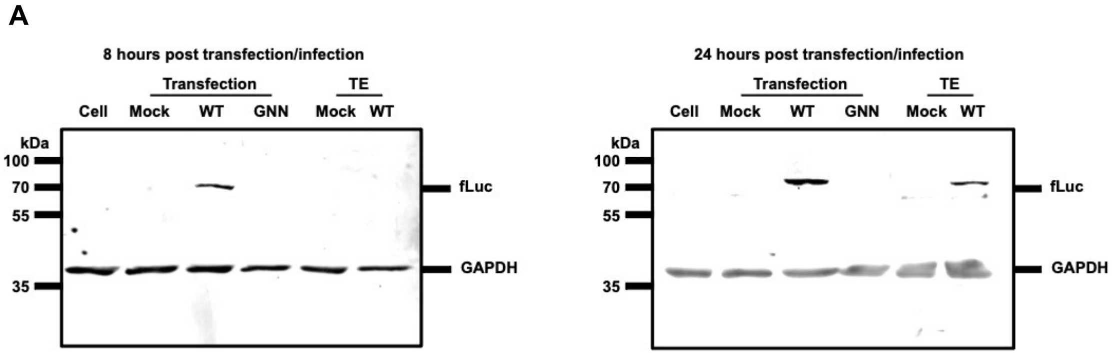


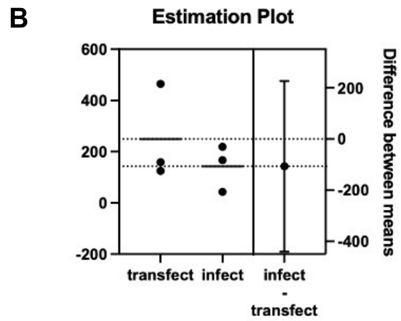


## Figure S6. Investigation of luciferase production

Anti-luciferase western blot of cell lysates at 6 and 24-hours post transfection with EVA71 luciferase replicon or infection with EVA71 P1-luciferase TE particles alongside cell only, mock or cognate GNN control. **A)** Blots dual probed for luciferase and GAPDH as loading control. Approximate molecular weights indicated. **B)** The difference between band intensities was determined at 24 hours, an no significant difference was found (n = 3).

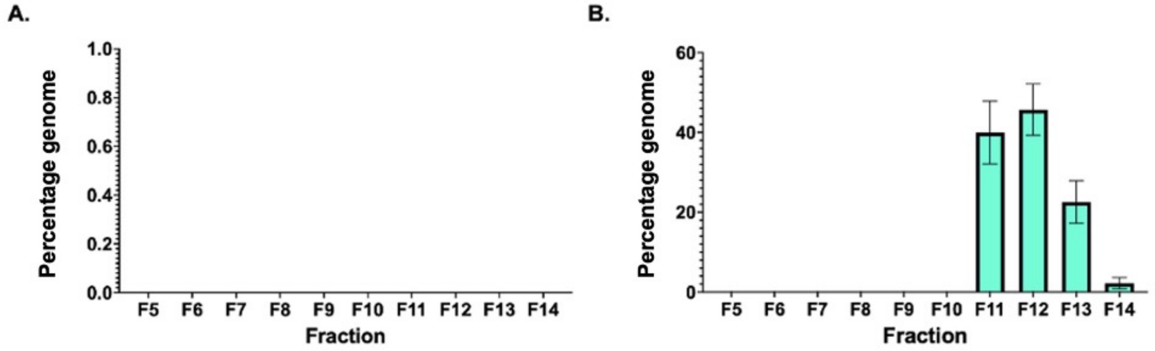


**Figure S7. Characterisation of TE particles and comparison to EVA71 virus particles P1-GFP GNN control**

Samples of P1-GFP and P1-GFP GNN TE particles were recovered directly from *in vitro* transcribed RNA electroporated into HeLa cells and separated on 15-45% sucrose gradients. Samples were collected top-down in 17 1mL fractions. **A)** P1-GFP GNN **B)** P1-GFP fractions 5-14 were assessed by RTqPCR for presence of genomic RNA and quantified relative to a titrated sample produced in the identical manner. Genome content is presented as percentage genome, graphed mean and SEM (n = 3). Assays performed in triplicate, graphed mean and SEM (n = 3).
